# Prof. Michele Baccarani. August 16, 1942 to December 20, 2021: a gifted life in haematology

**DOI:** 10.1038/s41375-022-01525-0

**Published:** 2022-02-28

**Authors:** Giuseppe Saglio, Gianantonio Rosti, Rüdiger Hehlmann, Andreas Hochhaus, Robert Peter Gale

**Affiliations:** 1 Department of Clinical and Biological Sciences, San Luigi University Hospital, University of Turin, Torino, Italy; 2 IRCCS/IRST ‘Dino Amadori’, Meldola, Italy; 3 Medical Faculty of Mannheim, University of Heidelberg, Mannheim, Germany; 4 University of Jena, Jena, Germany; 5 Department of Immunology and Inflammation, Hammersmith Hospital, London, UK

**Keywords:** Health sciences, Molecularly targeted therapy

**Figure:**
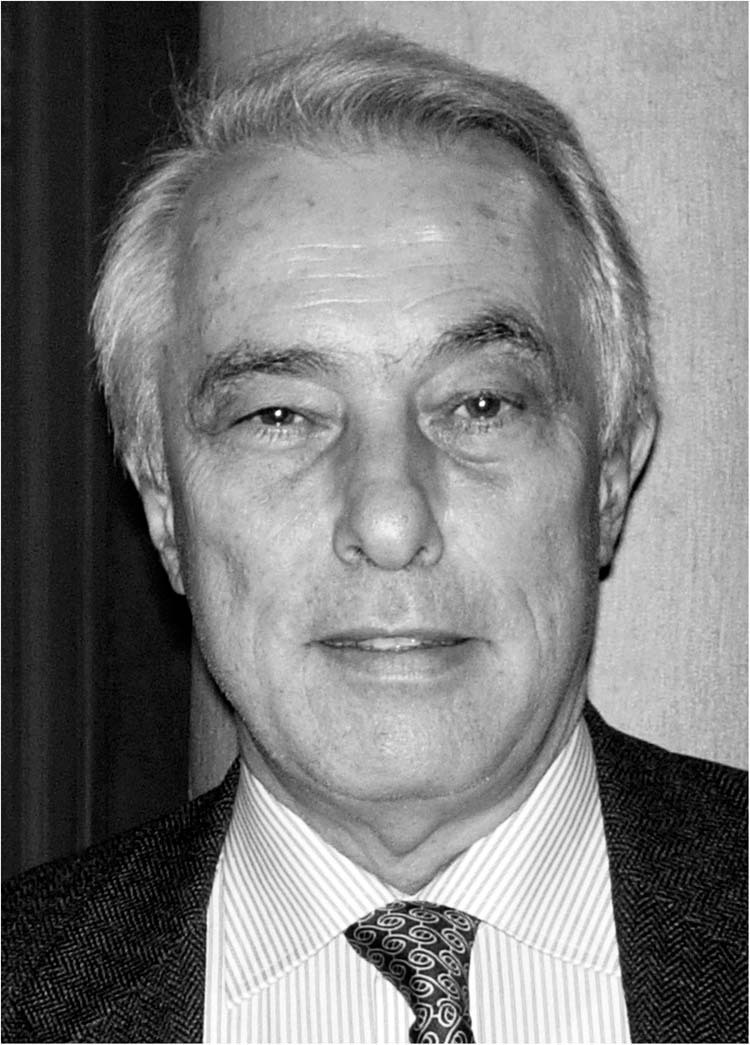
Prof. Michele Baccarani (courtesy of Prof. Gianni Pizzolo)

Italy lost one of its most outstanding hematologists, the international CML-community lost a leader. Prof. Michele Baccarani died on December 20, 2021, at age 79, in his beloved Bologna where he was born on August 16, 1942, into a professional family. (His father was a renowned otolaryngologist.) Prof. Baccarani received his MD degree at the University of Bologna School of Medicine in 1966 and did his thesis at the Institute of Electronic Microscopy of the University of Bologna. Prof. Baccarani was always greatly attracted to the study of blood cells and especially to the possibility of translating new technologies and biological advances to the clinic (now renamed “translational medicine” as if it is something entirely new*)*. As a post-doctoral student, he worked in the Division of Internal Medicine of the Sant’Orsola-Malpighi University Hospital of Bologna directed by Prof. Bruno Magnani. The haematology section was organized and directed by a brilliant young professor, Sante Tura. Prof. Baccarani did his specialization in haematology at the University of Modena in 1969 and, after marrying Barbara and the birth of his son Umberto (now a renowned surgeon in Udine), moved to the State Serum Institute and Rigshospialet in Copenhagen in 1971 as a research fellow with Profs. Sven-Aage Killmann and Nicole Muller-Berat, founding editors of *Leukemia*.

Between 1972 and 1986, Prof. Baccarani became the primary coworker of Tura. Their collaboration was the core of the Bologna Haematology Centre. His academic career took off in 1979 when he became Professor of Cancer Chemotherapy at the University of Chieti in central Italy. In 1986, he moved to Trieste as Professor of Haematology at the local university. In 1987, he took the chance to create a strong haematology group as Head of the Division of Hematology and the Department of Bone Marrow Transplantation at Udine University Hospital.

Despite increasing administrative and teaching responsibilities Prof. Baccarani never lost his passion for CML. He established important international collaborations. Particularly relevant was the one with Prof. Joseph Sokal in 1983 at Duke University in the US where he cooperated on the Sokal risk score becoming its doyen after Prof. Sokal’s death. Prognostic scores for CML were one of his passions when he later cooperated with Prof. Jörg Hasford in developing the Euro risk score and, again with Profs. Hasford and Markus Pfirrmann, in developing the more recent EUTOS and ELTS scores.

Prof. Baccarani was one of the founders and one of the most active members of the Italian Study Group on CML that later became the GIMEMA CML Working Party, a multi-center cooperative study group whose first leader was Prof. Sante Tura and which included more than 80 centers. This collaboration allowed Italian hematologists to participate in clinical trials and access advanced molecular technologies such as measurable residual disease testing. The group became internationally prominent in 1994 when they reported in a randomized controlled trial superiority of interferon-alpha over conventional chemotherapy. GS, GR, G. Martinelli, F. Pane and many others worked with him in this group. After Prof. Tura’s retirement, Michele became Chairman of the GIMEMA CML Working Party, a position he held for many years.

In 1993, Prof. Tura with Prof. Baccarani’s help invited the leading European clinicians interested in interferon and CML to a meeting in Venezia which started the annual workshops of the European investigators in CML (EI-CML) and which had a lasting impact on cooperative CML research across Europe and became the nucleus of the future European Leukemia Net (ELN).

When Prof. Tura retired in 2000, Baccarani succeeded him as Prof. of Hematology at the Alma Mater Studiorum, Università di Bologna, one of the most important Italian Universities and the oldest of the western world and Head of the Institute of Hematology and Medical Oncology and Department of Hematology and Oncology “L. and A. Seràgnoli” until his retirement in 2012. From 2001 to 2007, he served as Vice-Dean of the Faculty.

During his long, distinguished career Prof. Baccarani was President of the Italian Society of Experimental Haematology 1994–1995 and of the Italian Society of Haematology 2002–2003. As a Member and Chairman of the ELN-WP 4 on Chronic Myeloid Leukaemia, he led the development of the widely cited 2006, 2009 and 2013 ELN CML therapy recommendations putting him at center stage of international CML research and leading to appointment as chair of large leukemia meetings across Europe and as scientific head of the EUTOS CML-Registry from 2007 to 2015. The international CML-community remembers Michele Baccarani as a brilliant researcher of great intellectual honesty and an international leader in CML. European investigators on CML will pay tribute to him at the upcoming 28th EI-CML Workshop in Windsor, UK.

It is impossible to succinctly summarize Prof. Baccarani’s immense contributions to basic and clinical haematology in Italy and globally. He described his interests as chronic myeloid leukemia, acute leukemia, chemotherapy, haematopoietic cell transplantation, cell kinetics, stem cells, multi-drug resistance and molecular pathogenesis of leukemia. He served in multiple international committees and panels, most notably on the Boards of Directors of the ELN-Foundation and of the International CML Foundation.

Prof. Baccarani had a facile, lucid mind, always ready for a spirited critical debate but always in a constructive tone. He loved to quote Sir Peter Medawar who said: “Scientists who fall deeply in love with their hypothesis are proportionately unwilling to take no as an experimental answer”. Prof. Baccarani’s lodestone was always what is best for patients, especially those with CML. When he felt a discussion was becoming too complicated, abstract, remote or philosophical he loved to say: “We must think and behave as doctors” meaning we must focus on what is really helpful for the well-being of our patients. It is in this way we want to remember Michele Baccarani, a naturally born leader of the haematology community.

A true academic who believed in the Socratic method in his final days he formulated a list of important questions in CML therapy and challenged us to answer them in the coming years. His preliminary thoughts are available at Baccarani M, Bonifazi F, Soverini S, et al. 2002. Questions concerning tyrosine kinase-inhibitor therapy and transplants in chronic phase chronic myeloid leukaemia. *Leukemia*, in press.

Prof. Baccarani is survived by his wife Barbara, his son Umberto and his daughter Claudia and by three grandchildren to whom he wanted to transmit his love for life and sailing in the Mediterranean. We shall miss him as a scientist and as a friend.

